# Recurrent Self‐Limiting Acute Pancreatitis: Intraductal Papillary Mucinous Neoplasm Requiring Surgery

**DOI:** 10.1002/kjm2.70073

**Published:** 2025-06-30

**Authors:** Chia‐Hsin Chang, Yu‐Chun Ma, Shih‐Chang Chuang, Chih‐Wen Wang

**Affiliations:** ^1^ Division of Hepatobiliary, Department of Internal Medicine Kaohsiung Medical University Hospital, Kaohsiung Medical University Kaohsiung Taiwan; ^2^ Department of Internal Medicine Kaohsiung Municipal Siaogang Hospital, Kaohsiung Medical University Kaohsiung Taiwan; ^3^ Department of Pathology Kaohsiung Medical University Hospital, Kaohsiung Medical University Kaohsiung Taiwan; ^4^ Division of General and Digestive Surgery, Department of Surgery Kaohsiung Medical University Hospital, Kaohsiung Medical University Kaohsiung Taiwan

A 70‐year‐old woman with Type 2 diabetes mellitus who denied alcohol or tobacco use presented with mild, dull epigastric pain. The pain was relieved by the knee‐chest position and accompanied by nausea and vomiting, and she visited our emergency department after each episode. Significant increases in amylase (up to 4059 IU/L) and lipase (up to 6138 IU/L) levels recorded during over 10 visits for episodes of acute pancreatitis (Figure 1A). Interestingly, both amylase and lipase levels returned to normal within 2 days after each episode without any complications, and her epigastric pain improved spontaneously. She was subsequently discharged without issues.

**FIGURE 1:**
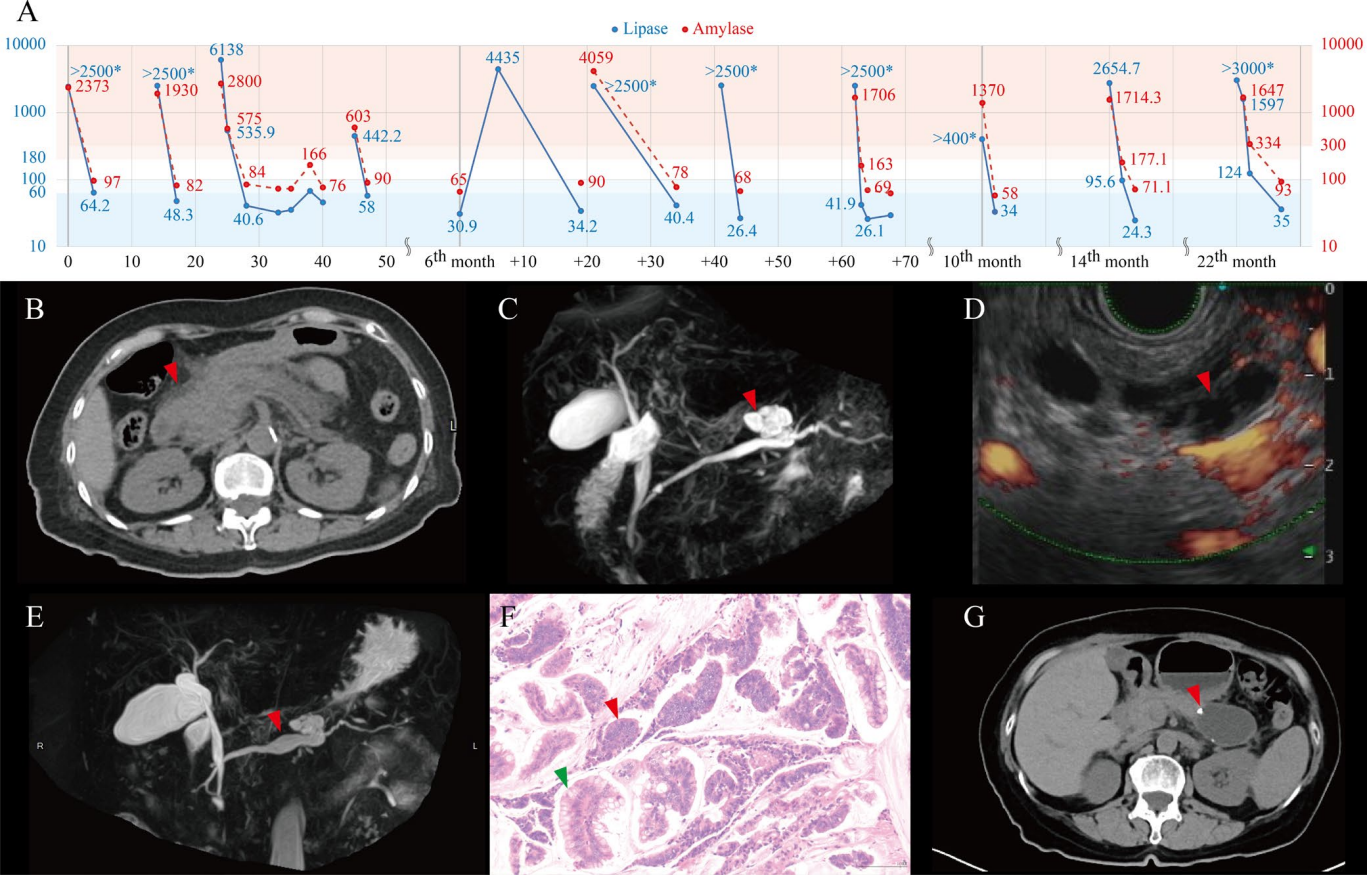
(A) Serial measurements of serum amylase (normal range < 100 IU/L) and lipase (normal range < 60 IU/L) levels over time. (B) Initially, the computed tomography showed pancreatic swelling accompanied by peri‐pancreatic infiltration and surrounding fat stranding (arrowhead). (C) Magnetic resonance cholangiopancreatography (MRCP) demonstrated a cystic neoplasm in the pancreatic body (arrowhead). (D) Endoscopic ultrasound identified well‐defined anechoic cystic lesions within the pancreatic body (arrowhead). (E) Follow‐up MRCP at 14 months post‐diagnosis revealed progressive enlargement of the cyst and dilation of the main pancreatic duct (arrowhead). (F) The patient underwent laparoscopic subtotal pancreatectomy. Histopathological examination confirmed intraductal proliferation of tall columnar (green arrowhead) and cuboidal mucin‐producing epithelial cells (red arrowhead), as visualized on Hematoxylin and Eosin (H&E) staining. (G) Postoperative abdominal CT 2 months after surgery showed a fluid collection consistent with seroma or biloma near the surgical clips at the resection site (arrowhead).

Computed tomography (CT) during the first episode of pancreatitis revealed pancreatic swelling with localized infiltration (Figure 1B). One month after the initial presentation, magnetic resonance cholangiopancreatography (MRCP) showed a cystic neoplasm in the pancreatic body connected to the main pancreatic duct (Figure 1C). Endoscopic ultrasonography (Figure 1D) with a through‐the‐needle biopsy from the stomach was performed in the 10th month, which revealed cystic lesions in the pancreatic body and elevated cystic carcinoembryonic antigen level, but no signs of malignancy. The procedure was complicated by acute pancreatitis, which resolved spontaneously within 2 days. CT and MRCP in the 14th month showed an enlarging cyst and dilation of the main pancreatic duct (Figure 1E). A surgical intervention was ultimately considered necessary, and laparoscopic subtotal pancreatectomy was performed in the 23rd month. Immunohistochemical staining of the specimen revealed both intestinal and pancreato‐biliary type epithelium. A pathological examination confirmed intraductal papillary mucinous neoplasm (IPMN) with low‐grade dysplasia (Figure 1F). Postoperative recovery was complicated by a grade A pancreatic fistula (Figure 1G), which was managed with retained drainage. Her condition gradually improved, and as of 2 years post‐surgery she has not experienced any further acute pancreatitis attacks.

In patients with acute pancreatitis, an abdominal examination may reveal Grey–Turner's sign or Cullen's sign in severe cases indicating retroperitoneal hemorrhage, while guarding or rebound tenderness may be present in more advanced cases such as necrotizing pancreatitis or secondary peritonitis. The differential diagnosis of acute pancreatitis is extensive and should be approached systematically, encompassing pancreatic, biliary, gastrointestinal, hepatic, cardiovascular, metabolic, toxic, infectious, and miscellaneous causes.

Guidelines vary in their threshold for recommending surgery [1]. The International Association of Pancreatology (IAP) [2] and European [3] guidelines adopt a more aggressive stance, considering that even small enhancing nodules (< 5 mm) are potential indicators for resection. The American College of Gastroenterology (ACG) [4] and European [3] guidelines also incorporate biomarkers such as elevated CA 19‐9 levels and new‐onset diabetes into their risk stratification. In contrast, the American Gastroenterological Association (AGA) 2015 [5] guidelines take a more conservative approach, recommending a surgical intervention only when multiple high‐risk features are present.

Our patient met multiple AGA [5], IAP [2], and European [3] guideline criteria for surgical resection of IPMN, including cyst size > 3 cm, main pancreatic duct dilation > 10 mm, progressive cyst growth, and recurrent pancreatitis. The decision to operate was clinically sound and evidence‐based, especially considering the risk of malignant transformation in mixed‐type IPMN. This case also contributes to a growing body of evidence that while IPMN‐related pancreatitis is often mild, it is an important indication for surgery.

## Conflicts of Interest

The authors declare no conflicts of interest.

## Data Availability

The data that support the findings of this study are available from the corresponding author upon reasonable request.
